# CD19/CD22 CAR-T-cell cocktail therapy following autologous transplantation is an optimizing strategy for treating relapsed/refractory central nervous system lymphoma

**DOI:** 10.1186/s40164-024-00538-y

**Published:** 2024-10-13

**Authors:** Xiaoxi Zhou, Qiuxia Yu, Zigang Dai, Jue Wang, Chunrui Li, Liang Huang, Yicheng Zhang, Yang Cao

**Affiliations:** 1 Department of Hematology, Tongji Hospital, Tongji Medical College, Huazhong University of Science and Technology, Wuhan, Hubei China; 2 Immunotherapy Research Center for Hematologic Diseases of Hubei Province, Wuhan, Hubei China

**Keywords:** CNS lymphoma, CAR-T-cell therapy, ASCT, Adverse events, Efficacy

## Abstract

Relapsed/refractory (R/R) primary and secondary central nervous system lymphomas (PCNSL, SCNSL) are associated with short survival and represent an unmet need, requiring novel effective strategies. We retrospectively compared the safety and efficacy of CD19/22 CAR-T-cell therapy following ASCT (ASCT + CAR-T group), CD19/22 CAR-T-cell cocktail therapy (CAR-T group) and chemoimmunotherapy (CIT group) in treating R/R CNSL patients. Analysis of the differences in clinical characteristics among the three groups revealed that the median age in the CIT group was older than that in the ASCT + CAR-T group and CAR-T group, and the median number of prior lines of therapy in the CIT group was less than that in the other groups. Patients in the two CAR-T-therapy groups exhibited comparable incidences and severities of CRS and ICANS. Grade 4–5 CRS and ICANS were not observed in either CAR-T-cell therapy group. The incidence of Grade 3/4 hematological toxicity in the ASCT + CAR-T and CAR-T groups was greater than that in the CIT group. The ORR was 82.75% in the ASCT + CAR-T group, 60.00% in the CAR-T group and 58.83% in the CIT group. As of December 31, 2022, the median follow-up after therapy was 16.73 months (range, 0.67-42.00 months). The median durations of PFS and OS were not reached in the ASCT + CAR-T group. The median PFS in the CAR-T group was 4.72 months, and OS was not reached. In the CIT group, the median PFS and OS were 6.63 months and 16.77 months, respectively. The 2-year PFS rate of patients in the ASCT + CAR-T group (65.52%) was significantly greater than that of patients in the CAR-T group (30.00%, *P* = 0.0321) and CIT group (23.53%, *P* = 0.0043). Our results support the development of CAR-T-cell therapy for R/R CNSL. With the durability of remission and low toxicity, ASCT combined with CAR-T-cell therapy appears to be a more effective and safer treatment option for primary and secondary R/R CNS lymphoma.

**Supplementary Information:**

The online version contains supplementary material available at 10.1186/s40164-024-00538-y.

To the editor,

Relapsed/refractory central nervous system (CNS) lymphoma, whether primary or secondary, is associated with poor prognosis using currently available treatment modalities [1, 2]. Chimeric antigen receptor (CAR)-T-cell therapy is a promising option for the treatment of CNSL owing to its high short-term remission rate and controllable side effects [3]. However, durable remission is limited [4, 5]. Thus, pursuing a higher response rate and durable remission are the main challenges for CAR-T-cell therapy in CNSL patients.

Our previous studies indicated that CAR-T-cell therapy combined with autologous hematopoietic stem cell transplantation (ASCT) may be a better treatment strategy, especially for R/R CNSL [6–8]. Herein, based on our previous clinical trials, the safety and efficacy of three treatment modalities were retrospectively compared.

From September 2019 to December 2021, a total of 71 patients were diagnosed with R/R CNSL at Tongji Hospital in Wuhan. Fifty-six patients were enrolled in this retrospective observational cohort study, including 29 patients from one clinical trial of CD19/22 CAR-T-cell therapy following ASCT (ASCT + CAR-T group), 10 patients from another clinical trial of CD19/22 CAR-T-cell cocktail therapy (CAR-T group), and 17 patients who received chemoimmunotherapy (CIT group) (Fig. 1A) [6, 9]. Methods and materials of this study can be found in the supplementary material.

**Fig. 1:**
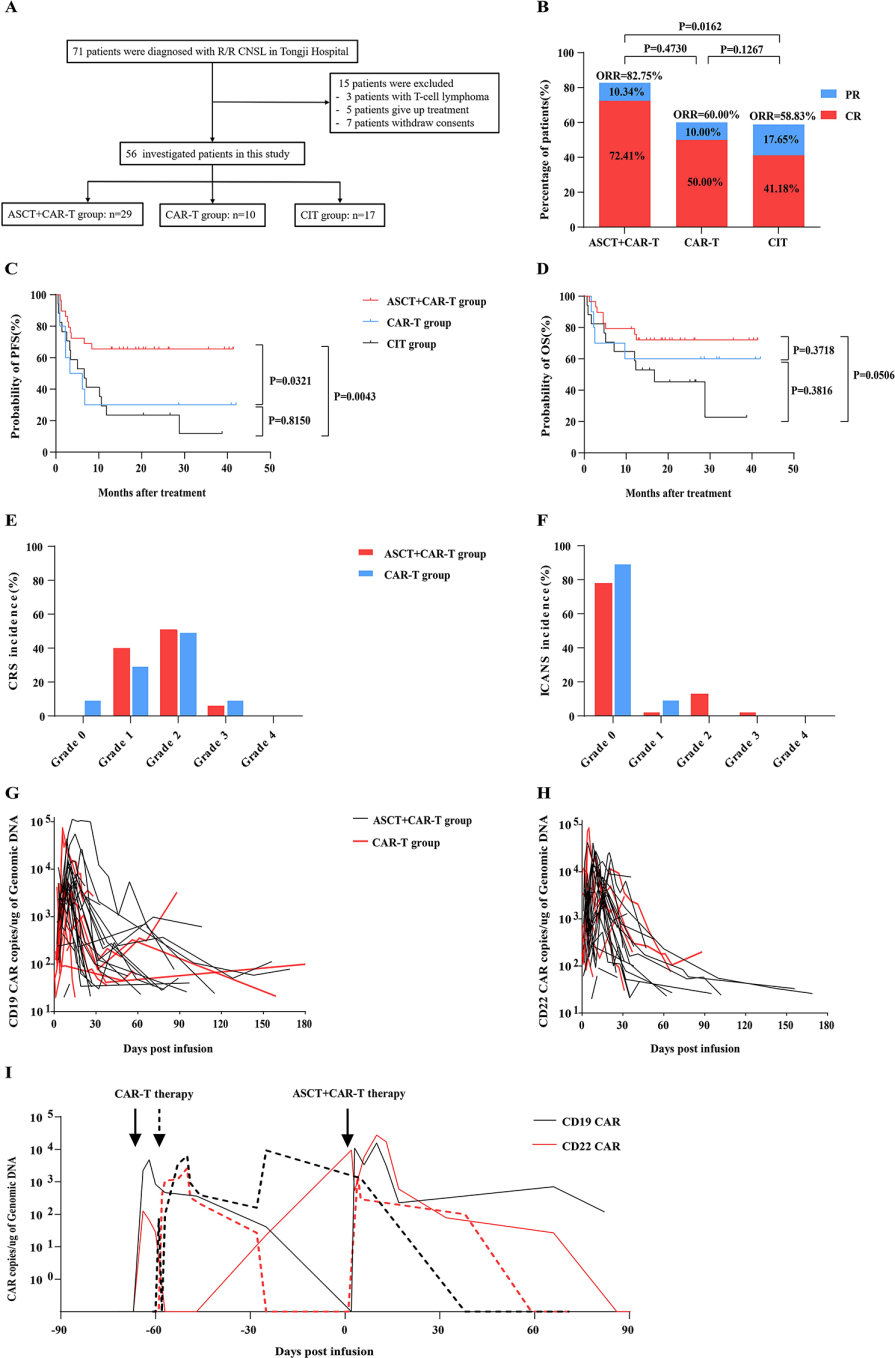
Patient selection and outcomes in all patients as well as CRS, ICANS, and the cellular kinetics of the CD19 and CD22 CAR transgenes in patients who received ASCT + CAR-T/CAR-T therapy. (**A**) Patient selection. (**B**) Response to therapy in all patients. In the ASCT + CAR-T group, the ORR was 82.75%, including 21 patients with CR and 3 patients with PR. A total of 95.2% (20/21) of patients who achieved CR maintained durable remission by the study cut-off. The ORR was 60.00% in the CAR-T group (6/10, 5 CR and 1 PR). In the CIT group, the ORR was 58.83% (10/17, 7 CR and 3 PR); (**C**) PFS and (**D**) OS in all patients. As of December 31, 2022, the median follow-up time was 16.73 months (range, 0.67-42.00 months). The median durations of PFS and OS were not reached in the ASCT + CAR-T group. The median PFS in the CAR-T group was 4.72 months, and OS was not reached. In the CIT group, the median PFS and OS were 6.63 months and 16.77 months, respectively. The red lines represent patients who received CD19/22 CAR-T-cell therapy combined with ASCT, the blue lines represent those who received CD19/22 CAR-T-cell cocktail therapy, and the black lines represent those who received chemoimmunotherapy; (**E**) The incidence of CRS and (**F**) ICANS in patients who received ASCT + CAR-T-cell/CAR-T-cell therapy. A total of 93.1% of patients in the ASCT + CAR-T group and 80.0% of patients in the CAR-T group experienced low-grade CRS (grade 1–2). Two patients in the ASCT + CAR-T group (6.9%) and one patient in the CAR-T group (10.0%) had grade 3 CRS. A total of 20.7% of patients in the ASCT + CAR-T group and 10.0% in the CAR-T group experienced ICANS. Grade 4–5 CRS and ICANS were not observed. The red bars represent those who received CD19/22 CAR-T-cell therapy combined with ASCT, and the blue bars represent those who received CD19/22 CAR-T-cell cocktail therapy. Copies of CD19 (**G**) and CD22 (**H**) CAR transgenes in the peripheral blood of patients who received ASCT + CAR-T-cell/CAR-T-cell therapy; (**I**) Copy numbers of CD19 and CD22 CAR transgenes in peripheral blood after CAR-T-cell therapy and after ASCT + CAR-T-cell therapy in the two patients in the ASCT + CAR-T group. Two patients who had previously received CAR-T-cell therapy and subsequently relapsed or had PD within three months were enrolled in the ASCT + CAR-T group (not included in the CAR-T group). Although the two patients in the ASCT + CAR-T-cell group received a second CAR-T-cell infusion, both the CAR-CD19 + and CAR-CD22 + T-cell populations still expanded well. At the 3-month assessment in the ASCT + CAR-T group, one patient achieved CR and was still alive at the day cut-off, with an OS of 20.97 months. Another patient maintained SD for 2 months and died of quickly progressive disease at 2.73 months

The patients in the ASCT + CAR-T group and CAR-T group were younger than those in the CIT group. Patients receiving CAR-T-cell therapy were more heavily pretreated than those receiving chemoimmunotherapy. In the ASCT + CAR-T group, two patients relapsed after CAR-T-cell therapy. Two patients in the CAR-T group and three patients in the CIT group underwent ASCT. 51.7%, 60.0% and 52.9% of patients in the ASCT + CAR-T group, CAR-T group, and CIT group, respectively, had disease progression (PD), and the remaining patients achieved stable disease (SD) without clinical improvement after their last-line therapy (Table 1). Within the ASCT + CAR-T group, 9 patients received BEAM and 20 patients underwent TBC as their conditioning regimen (Supplementary Table 1).

**Table 1:** Baseline characteristics of all patients

Characteristics	ASCT + CAR-T group, No. (%) ^#^ (*n* = 29)		CAR-T group, No. (%) (*n* = 10)	CIT group, No. (%) (*n* = 17)	*P* Value
Age, median (range), years		42(23–66)	38.5(18–66)	62(26–75)	**0.0060**
≤ 60		25(86.2)	9(90.0)	8(47.1)	
>60		4(13.8)	1(10.0)	9(52.9)	
Sex					0.3508
Male		14(48.3)	3(30.0)	10(58.8)	
Female		15(51.7)	7(70.0)	7(41.2)	
Diagnosis					**0.0051**
Primary CNS lymphoma		8(27.6)	0	10(58.8)	
Secondary CNS lymphoma		21(72.4)	10 (100.0)	7(41.2)	
Histology					
Diffuse large B-cell lymphoma		29(100.0)	10(100.0)	17(100.0)	
ECOG					0.3217
0–1		12(41.4)	5(50.0)	4(23.5)	
2–3		17(58.6)	5(50.0)	13(76.5)	
Genetics					
TP53 deletion/mutation		5(22.7, *n* = 22)	2(40.0, *n* = 5)	3(25.0, *n* = 12)	0.7257
MYD88^L265P^CD79b^wt^		7(31.8, *n* = 22)	0(0, *n* = 5)	3(25.0, *n* = 12)	0.3384
MYD88^L265P^CD79b^mut^		3(13.6, *n* = 22)	0(0, *n* = 5)	1(8.3, *n* = 12)	0.6400
Double-hit rearrangement		2(12.5, *n* = 16)	1(25.0, *n* = 4)	2(20.0, *n* = 10)	0.7866
Sites of disease before treatment					0.7408
CNS		25(86.2)	7(70.0)	17(100.0)	
IOL		2(6.9)	0	0	
CSF		4(13.8)	3(30.0)	3(17.6)	
Systemic disease		8(27.6)	3(30.0)	5(29.4)	
Remission status at inclusion					0.0762
Relapse		16(55.2)	9(90.0)	8(47.1)	
Refractory		13(44.8)	1(10.0)	9(52.9)	
Prior lines of therapy, median (range)		3(2–7)	5(3–9)	2(1–2)	<0.0001*
Disease status before treatment					0.9009
SD		14(48.3)	4(40.0)	8(47.1)	
PD		15(51.7)	6(60.0)	9(52.9)	

ASCT, autologous stem cell transplantation; CAR-T, chimeric antigen receptor T cells; CIT, chemoimmunotherapy; ECOG, Eastern Cooperative Oncology Group; CNS, central nervous system; CSF, cerebrospinal fluid; IOL, intraocular lymphoma; SD, stable disease; PD, progressive disease

^**#**^ Data represents No. (%) of patients unless otherwise identified as median (range)

*The patients in ASCT + CAR-T group and CAR-T group had received more prior lines of treatment than CIT group (*P* < 0.0001; *P* < 0.0001). And the patients in CAR-T group had received more prior lines of treatment than ASCT + CAR-T group (*P* = 0.0065)

Bold font :*P* Value < 0.05

The overall response rate (ORR) and complete response (CR) rate of the patients in the ASCT + CAR-T group (82.75%, 72.41%) were significantly greater than those of the patients in the CIT group (58.83%, 41.12%) (*P* = 0.0162, *P* = 0.0361) (Fig. 1B). The 2-year progression-free survival (PFS) rate of patients in the ASCT + CAR-T group was significantly greater than that of patients in the CAR-T group and CIT group (65.52% vs. 30.00%, *P* = 0.0321; 65.52% vs. 23.53%, *P* = 0.0043, respectively) (Fig. 1C). The 2-year overall survival (OS) rate of the patients in the ASCT + CAR-T group also exhibited an increasing trend compared to that of the patients in the CIT group (72.10% vs. 45.38%; *P* = 0.0506) (Fig. 1D).

In order to mitigate the heterogeneity, we performed subgroup analyses on the response and long-term survival of patients under 60 years old, with SCNSL, respectively (Supplementary Tables 2 and 3). The response and long-term survival of patients under 60 years old, with SCNSL align with the trend of observed in all patients.

According to both the univariate and multivariate analyses, PD before treatment (HR: 3.335, 95% CI, 1.014 to 10.259; *P* = 0.047) and more prior lines of therapy (HR: 1.342, 95% CI, 1.041 to 1.729; *P* = 0.023) were associated with worse PFS. PFS in the ASCT + CAR-T group exhibited a better trend than that in the CAR-T group (Supplementary Table 4). The PFS of patients with PD before treatment and the OS of patients with an ECOG score ≥ 2 in the ASCT + CAR-T group were superior to those in the CAR-T group (*P* = 0.0218, *P* = 0.0136) (Supplementary Fig. 1). In the ASCT + CAR-T group, factors such as age, disease status before treatment, conditioning regimen, primary or secondary CNS lymphoma, presence of systemic disease, high-risk genetic abnormalities and chemosensitivity did not have a significant impact on PFS and OS (Supplementary Fig. 2). To further investigate the efficacy of ASCT and CAR T-cells in both the CNS and systemic organs, subgroup analyses were conducted to compare PFS or OS in patients with or without systemic disease in ASCT + CAR-T/CAR-T group (Supplementary Fig. 3). Our analysis revealed no statistically significant difference in PFS and OS between patients with secondary CNS lymphoma with or without systemic disease in both ASCT + CAR-T group and CAR-T group.

All adverse events (AEs) that occurred within 30 days after therapy are summarized in Supplementary Table 5. The incidence of grade 3–4 hematological toxicity in the ASCT + CAR-T and CAR-T groups was greater than that in the CIT group. A total of 41.4% of patients in the ASCT + CAR-T group, 20.0% of patients in the CAR-T group and 11.8% of patients in the CIT group experienced ≥ grade 3 infections. One patient in the CIT group died of serious infection at 27 days. Most patients in both CAR-T groups experienced cytokine release syndrome (CRS) (Fig. 1E, F). A total of 20.7% of patients in the ASCT + CAR-T group and 10.0% in the CAR-T group experienced CAR-T-cell-related encephalopathy syndrome (ICANS). Patients in the two CAR-T-therapy groups exhibited comparable incidences and severities of CRS and ICANS. Grade 4–5 CRS and ICANS were not observed. The most common late AEs occurring beyond 1 month after treatment were haematological toxicity and infections (Supplementary Table 6). Furthermore, the incidence of adverse events gradually decreased over time. During long-term follow-up, no patients experienced late neurotoxicity or secondary neoplasia.

Both CAR-CD19 + and CAR-CD22 + T cells expanded well (Fig. 1G, H). Interestingly, two patients who had previously received CAR-T-cell therapy and subsequently relapsed or had PD within three months were enrolled in the ASCT + CAR-T group (not included in the CAR-T group). Although the two patients in the ASCT + CAR-T group received a second CAR-T-cell infusion, both the CAR-CD19 + and CAR-CD22 + T-cell populations still expanded well (Fig. 1I).

The median age in the CIT group was older than that in the other two groups, possibly due to the grouping mismatch caused by the preference for clinical trials. Increased knowledge of the molecular pathogenesis of CNSL has led physicians to prefer low-intensity chemotherapy combined with novel agents. Due to the low intensity of therapy, the patients in the CIT group had lower grade 3/4 hematological toxicity. Notably, combined CAR-T-cell therapy with ASCT was well tolerated, and the occurrence of severe CRS was even lower than that associated with CAR-T-cell therapy alone. These surprising findings might be attributable to myeloablative conditioning. On the one hand, tumor burden, which was shown in previous studies to be associated with severe CRS, might be alleviated by high-dose chemotherapy before ASCT [10]. On the other hand, the elimination of recipient myeloid cells by myeloablative conditioning may reduce the release of monocyte/macrophage-derived cytokines and reduce the incidence of severe CRS [11].

CAR-T-cell therapy is a promising option for the treatment of CNSL owing to a high short-term remission rate and controllable side effects. However, the high recurrence rate after remission must be addressed. We found a higher and durable response rate to ASCT combined with CAR-T-cell therapy. Although the number of prior lines in the ASCT+CAR-T group was significantly greater than that in the CIT group, the ORR, CR, PFS and OS rates were better than those in the CIT group. Compared to historical data (2-year PFS rates were approximately 20–50% for patients not reaching PR/CR before ASCT [2, 12], and 5 patients in our study received ASCT consolidation (mPFS was 3.43 months)), ASCT combined with CAR-T showed superior efficacy, even if the disease status before ASCT was SD or PD in most patients. Although ORR and CR between the CAR-T group and ASCT+CAR-T group were not significantly different, the PFS in the ASCT+CAR-T group was longer than that in the CAR-T group, similar to our previous report (ORR was 100% and mPFS was 3.07 months) [4], which suggested that CAR-T therapy in CNSL is effective but not long lasting.

With the durability of remission and low toxicity, ASCT combined with CAR-T-cell therapy appears to be a more effective and safer treatment option for R/R CNSL. A randomized controlled trial comparing combined ASCT with CAR-T-cell or CAR-T-cell therapy alone or ASCT alone is needed to confirm these encouraging results.

## Electronic supplementary material

Below is the link to the electronic supplementary material.

Supplementary Material 1

## Data Availability

No datasets were generated or analysed during the current study.
