# Supplementary material for: CD19/CD22 CAR-T-cell cocktail therapy following autologous transplantation is an optimizing strategy for treating relapsed/refractory central nervous system lymphoma
Source: Exp Hematol Oncol. 2024 Oct 13;13:100. doi: 10.1186/s40164-024-00538-y (PMC11471030; doi:10.1186/s40164-024-00538-y)
Supplement: Supplementary file 1 — Supplementary Material 1 [file 40164_2024_538_MOESM1_ESM.docx]

**Supplemental Materials**

This Supplemental Materials is provided by the authors for additional information about their work.

**CONTENTS:**

**Methods and Materials**

**Supplemental Tables**

Supplementary Table 1. Characteristics of CAR-T Treatment.

Supplementary Table 2. Baseline Characteristics, Response and Survival Outcomes in Patients Under 60 Years Old.

Supplementary Table 3. Baseline Characteristics, Response and Survival Outcomes in Patients Under 60 Years Old with Secondary CNS Lymphoma.

Supplementary Table 4. Univariate and Multivariate Cox Model for Predictor of PFS in patients who received ASCT+CART/CART therapy.

Supplementary Table 5. Adverse Events During Therapy Within 30 Days.

Supplementary Table 6. Long Term Adverse Events (Grade ≥3) Occurred in Patients.

**Supplemental Figures**

Supplementary Figure 1. Survival outcomes in patients received ASCT+CAR-T/CAR-T therapy.

Supplementary Figure 2. Subgroup analysis of PFS or OS in ASCT+CAR-T group.

Supplementary Figure 3. Subgroup analysis of PFS or OS in patients with/without systemic disease in ASCT+CAR-T/CAR-T group.

**Methods and Materials**

**Study design**

From September 2019 to December 2021, a total of 71 patients were diagnosed with relapsed/refractory CNSL in Tongji Hospital in Wuhan. Three patients with T-cell lymphoma were excluded, 5 patients refused further treatment, and 7 patients withdrew consent. Fifty-six patients were enrolled in this retrospective observational cohort study, including 29 patients from one clinical trial of CD19/22 CAR-T-cell therapy following ASCT (ASCT+CAR-T group, registry number ChiCTR-OPN-16009847), 10 patients from another clinical trial of CD19/22 CAR-T-cell cocktail therapy (CAR-T group, registry number ChiCTR-OPN-16008526), and 17 patients who received chemoimmunotherapy (CIT group) (Figure 1)^1, 2^. CNS involvement was an eligibility criterion for clinical trials of CD19/22 CAR-T-cell therapy following ASCT since the inclusion criteria were revised in August 2019. All the cases were adult patients with R/R CNS lymphoma who had previously received at least two-line treatments. The study was approved by the Ethics Committee of Tongji Hospital, Tongji Medical College, Huazhong University of Science and Technology, and all subjects provided written informed consent.

**Generation of CAR-T cells and treatment**

T cells were obtained from patients who received CAR-T therapy and stimulated, transfected, expanded and examined in vitro as previously described^2^. In the CAR-T group and ASCT+CAR-T group, the same third-generation CAR product, including murine anti-CD19 or anti-CD22 single-chain variable fragment (scFv), CD28, 4-1BB costimulatory domains and CD3-z signalling domain, was used. In addition, patients who received HDT-ASCT followed by CAR-T therapy underwent granulocyte colony-stimulating factor-primed autologous stem-cell collection. A minimum of 2×10^6^ CD34+ cells/kg is the standard eligibility parameter as previously described^1^.

For lymphodepletion, the patients received CAR-T therapy and were given an FC conditioning regimen (fludarabine 25 mg/m^2^ and cyclophosphamide 300 mg/m^2^ daily for 3 days, days -4 to -2) before CAR-T-cell infusion (CTI). Anti-CD19 and anti-CD22 CAR-T cells were infused separately on successive days from day zero.

For those who received ASCT followed by CAR-T therapy, a BEAM conditioning regimen (biscarmusitine 270 mg/(m^2^·d) on day -7, etoposide 200 mg/(m^2^·d) and cytarabine 400 mg/(m^2^·d) on days -6, -5, -4, -3, melphalan 140 mg/(m^2^·d) on day -2) or a TBC conditioning regimen (thiotepa 250 mg/(m^2^·d) on days -9, -8, -7, busulfan 3.2 mg/(kg·d) on days -6, -5, -4, and cyclophosphamide 60 mg/(kg·d) on days -3, -2) was given as myeloablative chemotherapy before autologous stem-cell infusion (SCI). Autologous haematopoietic stem cells were infused on day zero, and anti-CD19 and anti-CD22 CAR-T cells were infused separately on successive days from day two but for no more than 5 days.

In the CIT group, 64.7% (11/17) of the patients used salvage chemotherapy, including high-dose methotrexate-based and high-dose cytarabine-based chemotherapy. A total of 52.9% (9/17) of the patients used targeted drugs, including BTK inhibitors (29.4%, 5/17) and lenalidomide (23.5%, 4/17).

**Response and toxicity assessment**

We used contrast-enhanced brain magnetic resonance imaging (MRI) and cerebrospinal fluid (CSF) analysis to assess the response of CNS lymphoma and positron emission tomography/computed tomography (PET/CT) for systemic disease^3, 4^. Response evaluation was conducted monthly for the first 3 months after treatment and then at an interval of 2-3 months. The follow-up ended on December 31, 2022. Overall survival (OS) was calculated from the time of treatment to the last follow-up or death. Progression-free survival (PFS) was defined as the duration from treatment to any event: lymphoma progression, recurrence of disease and any cause-related mortality. The overall response rate (ORR) was calculated as the combined rates of complete response (CR) and partial response (PR).

Cytokine release syndrome (CRS) was graded according to the CAR-T-cell-therapy-associated TOXicity (CARTOX) Working Group Consensus^5^. CAR-T-cell-related encephalopathy syndrome (ICANS) and other adverse events were evaluated according to the Common Terminology Criteria for Adverse Events (CTCAE) version 5.0^6, 7^.

**Laboratory assessments**

Dual expression of CD19 and CD22 on malignant B cells was confirmed by flow cytometry or immunohistochemistry (IHC). A total of 157 lymphoma-related genes with mutation hot spots were assessed using next-generation sequencing (NGS) with NEXTSEQ 550 (Illumina, San Diego, CA, USA). TP53 deletion and double-hit rearrangement were determined by fluorescence in situ hybridization (FISH).

CAR copy numbers were detected by droplet digital polymerase chain reaction (ddPCR), and CD19+ B cells in the peripheral blood were analysed on a FACS Calibur (Becton Dickinson, San Jose, CA, USA) flow cytometer. Details were previously described^7^.

**Statistical analysis**

The analysis of categorical variables was performed using χ2 or Fisher’s exact test, and continuous variables used Student’s t test between two groups, while a one-way analysis of variance (ANOVA) was applied to compare more than two groups. Survival analyses were analysed using the Kaplan‒Meier method and compared with the use of the log-rank test. Multivariate survival analyses were performed using the Cox proportional hazards model (Cox regression). Variables with P < 0.1 in univariate analysis were included in a multivariate stepwise logistic analysis, and variables significant at P < 0.05 were retained in the final model. The 95% confidence interval (CI) was calculated by the Wilson method. Statistical analyses were performed using Statistical Package for the Social Sciences (SPSS) 22.0 software (SPSS Inc., Chicago, USA) and GraphPad Prism software (version 9.0). A two-tailed P value <0.05 was considered significant.

**References**

1. Cao Y，Xiao Y，Wang N，et al. CD19/CD22 Chimeric Antigen Receptor T Cell Cocktail Therapy following Autologous Transplantation in Patients with Relapsed/Refractory Aggressive B Cell Lymphomas. Transplant Cell Ther. 2021;27:910.e911-910.e911. doi:10.1016/j.jtct.2021.08.012

2. Wang N，Hu X，Cao W，et al. Efficacy and safety of CAR19/22 T-cell cocktail therapy in patients with refractory/relapsed B-cell malignancies. Blood. 2020;135:17-27. doi:10.1182/blood.2019000017

3. Abrey L E，Batchelor T T，Ferreri A J，et al. Report of an international workshop to standardize baseline evaluation and response criteria for primary CNS lymphoma. J Clin Oncol. 2005;23:5034-5043. doi:10.1200/jco.2005.13.524

4. Cheson B D，Fisher R I，Barrington S F，et al. Recommendations for initial evaluation, staging, and response assessment of Hodgkin and non-Hodgkin lymphoma: the Lugano classification. J Clin Oncol. 2014;32:3059-3068. doi:10.1200/jco.2013.54.8800

5. Lee D W，Santomasso B D，Locke F L，et al. ASTCT Consensus Grading for Cytokine Release Syndrome and Neurologic Toxicity Associated with Immune Effector Cells. Biol Blood Marrow Transplant. 2019;25:625-638. doi:10.1016/j.bbmt.2018.12.758

6. Neelapu S S，Tummala S，Kebriaei P，et al. Chimeric antigen receptor T-cell therapy - assessment and management of toxicities. Nat Rev Clin Oncol. 2018;15:47-62. doi:10.1038/nrclinonc.2017.148

7. Zeng C，Cheng J，Li T，et al. Efficacy and toxicity for CD22/CD19 chimeric antigen receptor T-cell therapy in patients with relapsed/refractory aggressive B-cell lymphoma involving the gastrointestinal tract. Cytotherapy. 2020;22:166-171. doi:10.1016/j.jcyt.2020.01.008

**Supplementary Table 1. Characteristics of CAR-T Treatment.**

| Events | ASCT+CAR-T group,  No. (%) ^#^ (n= 29) | CAR-T group,  No. (%) (n= 10) | *P* Value |
| --- | --- | --- | --- |
| Conditioning regimen before ASCT+CAR-T/CAR-T |  |  |  |
| BEAM | 9(31.0) | / |  |
| TBC | 20(69.0) | / |  |
| FC | / | 10(100) |  |
| CAR-T cell dose, ×10^6^ cells/kg, median (range) |  |  |  |
| CD19 CAR-T cells | 4.00(0.75-5.75) | 4.11(2.23-8.21) | 0.0648 |
| CD22 CAR-T cells | 4.00(1.13-5.84) | 4.00(1.47-5.51) | 0.2437 |

ASCT, autologous stem cell transplantation; CAR-T, chimeric antigen receptor T cells; BEAM, bis-carmusitine, etoposide, cytarabine and melphalan; TBC, thiotepa, busulfan and cyclophosphamide; FC, fludarabine and cyclophosphamide.

**^#^**Data represents No. (%) of patients unless otherwise identified as median (range).

**Supplementary Table 2. Baseline Characteristics, Response and Survival Outcomes in Patients Under 60 Years Old.**

| Characteristics | ASCT+CAR-T group,  No. (%) ^#^ (n= 25) | | CAR-T group,  No. (%) (n= 9) | CIT group,  No. (%) (n= 8) | *P* Value |
| --- | --- | --- | --- | --- | --- |
| Age, median (range), years | | 42(23-60) | 34(18-60) | 45.5(26-59) | 0.5081 |
| Sex | |  |  |  | 0.4741 |
| Male | | 11(44.0) | 3(33.3) | 5(62.5) |  |
| Female | | 14(56.0) | 6(66.7) | 3(37.5) |  |
| Diagnosis | |  |  |  | **0.0021** |
| Primary CNS lymphoma | | 6(24.0) | 0 | 6(75.0) |  |
| Secondary CNS lymphoma | | 19(76.0) | 9 (100.0) | 2(25.0) |  |
| ECOG | |  |  |  | 0.4094 |
| 0-1 | | 12(48.0) | 5(55.6) | 2(25.0) |  |
| 2-3 | | 13(52.0) | 4(44.4) | 6(75.0) |  |
| Genetics | |  |  |  |  |
| TP53 deletion/mutation | | 5(27.8, n=18) | 2(40.0, n=5) | 1(14.3, n=7) | 0.6021 |
| MYD88^L265P^CD79b^wt^ | | 4(22.2, n=18) | 0(0, n=5) | 2(28.6, n=7) | 0.4433 |
| MYD88^L265P^CD79b^mut^ | | 1(5.6, n=18) | 0(0, n=5) | 0(0, n=7) | 0.7083 |
| Double-hit rearrangement | | 1(7.1, n=14) | 1(25.0, n=4) | 1(25.0, n=4) | 0.5020 |
| Sites of disease before treatment | |  |  |  | 0.8304 |
| CNS | | 17(68.0) | 7(77.8) | 6(75.0) |  |
| Systemic Disease | | 8(32.0) | 2(22.2) | 2(25.0) |  |
| Remission status at inclusion | |  |  |  | 0.2089 |
| Relapse | | 14(56.0) | 8(88.9) | 5(62.5) |  |
| Refractory | | 11(44.0) | 1(11.1) | 3(37.5) |  |
| Prior lines of therapy, median (range) | | 3(2-7) | 5(3-9) | 2(1-2) | **＜0.0001** |
| Disease status before treatment | |  |  |  | 0.4848 |
| SD | | 12(48.0) | 3(33.3) | 5(62.5) |  |
| PD | | 13(52.0) | 6(66.7) | 3(37.5) |  |
| CR rate (%) | | 72.0 | 44.4 | 50.0 | 0.2561**^*^** |
| ORR (%) | | 84.0 | 55.5 | 75.0 | 0.2277**^*^** |
| 2-year PFS rate (%) | | 68.0 | 22.2 | 37.5 | **0.0187^**^** |
| 2-year OS rate (%) | | 72.0 | 55.6 | 37.5 | 0.1410**^**^** |

ASCT, autologous stem cell transplantation; CAR-T, chimeric antigen receptor T cells; CIT, chemoimmunotherapy; ECOG, Eastern Cooperative Oncology Group; CNS, central nervous system; CSF, cerebrospinal fluid; IOL, intraocular lymphoma; SD, stable disease; PD, progressive disease; CR, complete response; ORR, overall response rate; PFS, progression-free survival; OS, overall survival.

**^#^**Data represents No. (%) of patients unless otherwise identified as median (range).

* The ORR and CR rate of the patients under 60 years old in the ASCT+CAR-T group was higher than that of the patients in the CAR-T group (P=0.4844 and P<0.001) and CIT group (P=0.0051 and P=0.0022).

** The PFS and OS of the patients under 60 years old in the ASCT+CAR-T group was higher than that of the patients in the CAR-T group (P=0.0085 and P=0.2737) and CIT group (P=0.0893 and P=0.0494).

Bold font :*P* Value <0.05.

**Supplementary Table 3. Baseline Characteristics, Response and Survival Outcomes in Patients Under 60 Years Old with Secondary CNS Lymphoma.**

| Characteristics | ASCT+CAR-T group,  No. (%) ^#^ (n= 19) | | CAR-T group,  No. (%) (n= 9) | CIT group,  No. (%) (n= 2) | *P* Value |
| --- | --- | --- | --- | --- | --- |
| Age, median (range), years | | 39(23-60) | 34(18-60) | 33.5(26-41) | 0.7203 |
| Sex | |  |  |  | 0.8671 |
| Male | | 8(42.1) | 3(33.3) | 1(50.0) |  |
| Female | | 11(57.9) | 6(66.7) | 1(50.0) |  |
| ECOG | |  |  |  | 0.2919 |
| 0-1 | | 11(57.9) | 5(55.6) | 0 |  |
| 2-3 | | 8(42.1) | 4(44.4) | 2(100.0) |  |
| Genetics | |  |  |  |  |
| TP53 deletion/mutation | | 1(7.7, n=13) | 2(40.0, n=5) | 0(0, n=2) | 0.1875 |
| MYD88^L265P^CD79b^wt^ | | 1(7.7, n=13) | 0(0, n=5) | 1(50.0, n=2) | 0.1232 |
| MYD88^L265P^CD79b^mut^ | | 1(7.7, n=13) | 0(0, n=5) | 0(0, n=2) | 0.7532 |
| Double-hit rearrangement | | 1(11., n=9) | 1(25.0, n=4) | 0(0, n=1) | 0.7350 |
| Sites of disease before treatment | |  |  |  | 0.1212 |
| CNS | | 11(57.9) | 7(77.8) | 0 |  |
| Systemic Disease | | 8(42.1) | 2(22.2) | 2(100.0) |  |
| Remission status at inclusion | |  |  |  | 0.2409 |
| Relapse | | 12(63.2) | 8(88.9) | 2(100.0) |  |
| Refractory | | 7(36.8) | 1(11.1) | 0 |  |
| Prior lines of therapy, median (range) | | 3(2-7) | 5(3-9) | 1(1) | **0.0023** |
| Disease status before treatment | |  |  |  | 0.3323 |
| SD | | 9(47.4) | 3(33.3) | 0 |  |
| PD | | 10(52.6) | 6(66.7) | 2(100.0) |  |
| CR rate (%) | | 68.4 | 44.4 | 50.0 | 0.4602**^*^** |
| ORR (%) | | 79.0 | 55.5 | 50.0 | 0.3680**^*^** |
| 2-year PFS rate (%) | | 63.2 | 22.2 | 0 | **0.0418^**^** |
| 2-year OS rate (%) | | 68.4 | 55.6 | 0 | 0.0580**^**^** |

ASCT, autologous stem cell transplantation; CAR-T, chimeric antigen receptor T cells; CIT, chemoimmunotherapy; ECOG, Eastern Cooperative Oncology Group; CNS, central nervous system; CSF, cerebrospinal fluid; IOL, intraocular lymphoma; SD, stable disease; PD, progressive disease; CR, complete response; ORR, overall response rate; PFS, progression-free survival; OS, overall survival.

**^#^**Data represents No. (%) of patients unless otherwise identified as median (range).

* The ORR and CR rate of the patients under 60 years old in the ASCT+CAR-T group was higher than that of the patients in the CAR-T group (P=0.2007 and P=0.2250) and CIT group (P=0.3606 and P=0.5991).

** The PFS and OS of the patients under 60 years old in the ASCT+CAR-T group was higher than that of the patients in the CAR-T group (P=0.0322 and P=0.4008) and CIT group (P=0.0571 and P=0.0139).

Bold font :*P* Value <0.05.

**Supplementary Table 4. Univariate and Multivariate Cox Model for Predictor of PFS in patients who received ASCT+CART/CART therapy.**

| Variable | Univariate analysis | | | Multivariate analysis^#^ | | |
| --- | --- | --- | --- | --- | --- | --- |
|  | *P* Value | HR | 95% CI | *P* Value | HR | 95% CI |
| Age, >60 years | 0.862 | 0.844 | 0.125-5.720 |  |  |  |
| Diagnosis, PCNSL | 0.246 | 0.356 | 0.062-2.043 |  |  |  |
| ECOG, ≥2 | 0.096 | 0.327 | 0.087-1.221 |  |  |  |
| Disease status before treatment, PD | **0.016** | 5.687 | 1.378-23.480 | **0.047** | 3.225 | 1.014-10.259 |
| Prior lines of therapy | **0.024** | 1.800 | 1.080-3.001 | **0.023** | 1.342 | 1.041-1.729 |
| Treatment, ASCT+CAR-T | 0.060 | 0.226 | 0.048-1.067 |  |  |  |

HR, hazard ratio; CI, confidence interval; PCNSL, primary central nervous system lymphoma; ECOG, Eastern Cooperative Oncology Group; PD, progressive disease; ASCT, autologous stem cell transplantation; CAR-T, chimeric antigen receptor–modified T cells.

^#^Multivariate analysis includes variables with *P* < 0.05 in univariate analysis.

Bold font: *P* Value <0.05.

**Supplementary Table 5. Adverse Events During Therapy Within 30 Days.**

| Events | ASCT+CAR-T group,  No. (%) ^#^ (n= 29) | | | CAR-T group,  No. (%) (n= 10) | | CIT group,  No. (%) (n= 17) | |
| --- | --- | --- | --- | --- | --- | --- | --- |
|  | Grade 1-2 | Grade ≥3 | | Grade 1-2 | Grade ≥3 | Grade 1-2 | Grade ≥3 |
| Any treatment-emergent adverse events | 28(96.6) | | 29(100) | 10(100) | 9(90.0) | 11(64.7) | 8(47.1) |
| Deaths due to treatment-emergent adverse events | 0 | | 0 | 0 | 0 | 0 | 0 |
| Neurotoxicity |  | |  |  |  |  |  |
| Tremor | 1(3.4) | | 0 | 0 | 0 | 0 | 0 |
| Dysphasia | 1(3.4) | | 0 | 0 | 0 | 0 | 0 |
| Hypersomnia | 4(13.8) | | 0 | 0 | 0 | 0 | 0 |
| Memory impairment | 2(6.9) | | 0 | 0 | 0 | 0 | 0 |
| Cognitive disturbance | 3(10.3) | | 0 | 1(10.0) | 0 | 1(5.9) | 0 |
| Seizure | 0 | | 1(3.4) | 0 | 0 | 0 | 0 |
| Depressed level of consciousness | 1(3.4) | | 0 | 0 | 0 | 0 | 0 |
| Hematological toxicity |  | |  |  |  |  |  |
| Neutropenia | 0 | | 29(100) | 1(10.0) | 9(90.0) | 3(17.6) | 8(47.1) |
| Anemia | 0 | | 29(100) | 3(30.0) | 7(70.0) | 3(17.6) | 7(41.2) |
| Thrombocytopenia | 0 | | 29(100) | 2(20.0) | 8(80.0) | 2(11.8) | 8(47.1) |
| Other toxicities |  | |  |  |  |  |  |
| Fever | 28(96.6) | | 1(3.4) | 9(90.0) | 0 | 10(58.8) | 0 |
| Infections | 3(10.3) | | 12(41.4) | 2(20.0) | 2(20.0) | 2(11.8) | 2(11.8) |
| Elevated ALT/AST | 2(6.9) | | 1(3.4) | 0 | 1(10.0) | 3(17.6) | 1(5.9) |
| Arrhythmias | 0 | | 1(3.4) | 0 | 1(10.0) | 0 | 0 |
| Heart failure | 0 | | 0 | 0 | 1(10.0) | 0 | 1(5.9) |
| Mucositis | 4(13.8) | | 10(34.5) | 3(30.0) | 1(10.0) | 3(17.6) | 0 |
| Emesis | 9(31.0) | | 2(6.9) | 4(40.0) | 0 | 4(23.5) | 0 |
| Nausea | 16(55.2) | | 1(3.4) | 6(60.0) | 0 | 8(47.1) | 0 |
| Hypothyroidism | 1(3.4) | | 0 | 1(10.0) | 0 | 0 | 0 |
| Upper gastrointestinal hemorrhage | 0 | | 4(13.8) | 0 | 0 | 0 | 0 |

ASCT, autologous stem cell transplantation; CAR-T, chimeric antigen receptor–modified T cells; CIT, chemoimmunotherapy; ALT, alanine transaminase; AST, aspartate transaminase.

**^#^**Data represents No. (%) of patients unless otherwise identified as median (range).

**Supplementary Table 6. Long Term Adverse Events (Grade ≥3) Occurred in Patients.**

| Events | from 1 month to 1 year | | | | |  | from 1 year to cut-off date | | |
| --- | --- | --- | --- | --- | --- | --- | --- | --- | --- |
|  | ASCT+CAR-T group  (n= 29) | CAR-T group  (n= 10) | | CIT group  (n= 15) |  | | ASCT+CAR-T group  (n= 21) | CAR-T group  (n= 6) | CIT group  (n= 10) |
| Hematological toxicity, No. (%) |  | |  |  |  | |  |  |  |
| Neutropenia | 5(17.2) | | 3(30.0) | 1(6.7) |  | | 0 | 0 | 0 |
| Anemia | 2(6.9) | | 3(30.0) | 0 |  | | 0 | 0 | 0 |
| Thrombocytopenia | 7(24.1) | | 3(30.0) | 2(13.3) |  | | 0 | 0 | 0 |
| Other toxicities, No. (%) |  | |  |  |  | |  |  |  |
| Infections | 13(44.8) | | 5(50.0) | 5(33.3) |  | | 3(14.3) | 2(33.3) | 2(20.0) |
| Cholecystitis | 2(6.9) | | 0 | 0 |  | |  |  |  |
| Heart failure | 1(3.4) | | 1(10.0) | 0 |  | | 1(4.8) | 0 | 0 |
| Upper gastrointestinal hemorrhage | 0 | | 1(10.0) | 0 |  | | 0 | 0 | 0 |

ASCT, autologous stem cell transplantation; CAR-T, chimeric antigen receptor–modified T cells; CIT, chemoimmunotherapy.


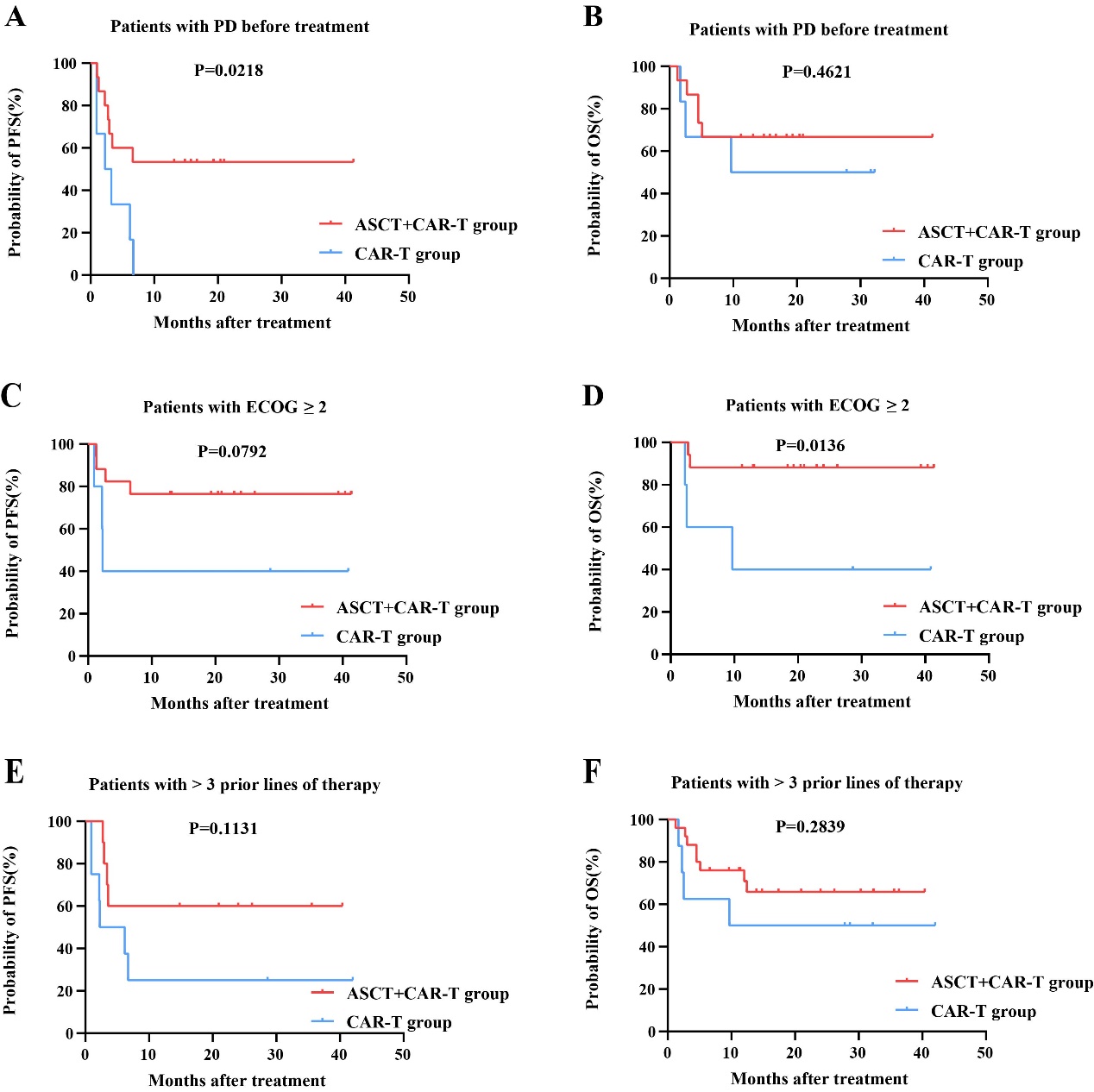


Supplementary Figure 1. Survival outcomes in patients received ASCT+CAR-T/CAR-T therapy. (A) PFS and (B) OS in patients with PD before treatment; (C) PFS and (D) OS in patients with an Eastern Cooperative Oncology Group (ECOG) score≥2; (E) PFS and (F) OS in patients with prior lines of therapy >3. PFS of patients with PD before treatment and OS of patients with ECOG ≥2 in the ASCT+CAR-T group were superior to CAR-T group. The red lines represent those who received CD19/22 CAR T-cell therapy combined with HDT-ASCT, and the blue lines represent those who received CD19/22 CAR T-cell cocktail therapy. PFS and OS of patients with PD before treatment, patients with ECOG ≥2, and with＞3 prior lines of therapy in the ASCT+CAR-T group were superior to CAR-T group.


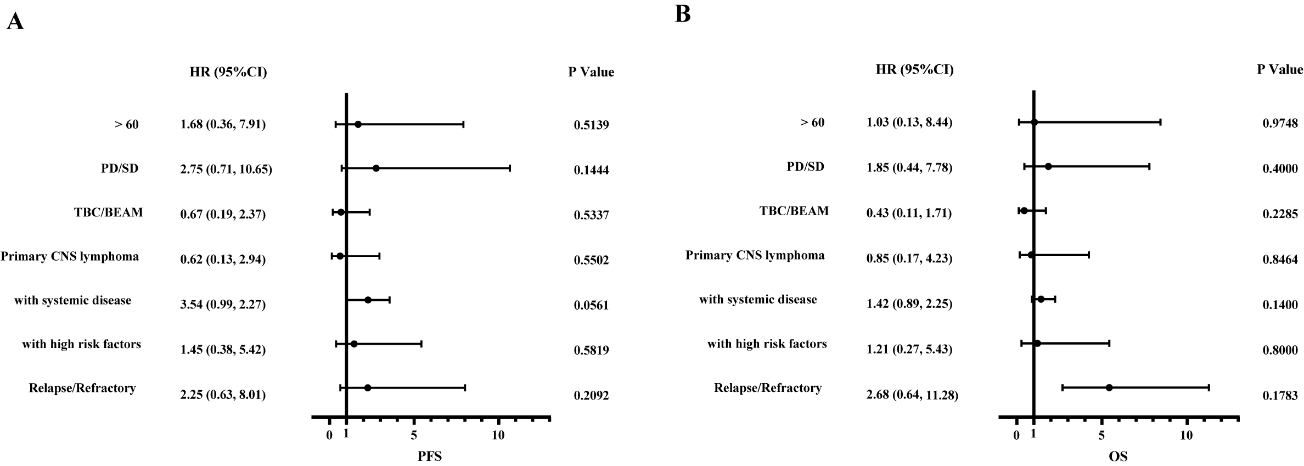


Supplementary Figure 2. Subgroup analysis of PFS or OS in ASCT+CAR-T group. (A) Subgroup analysis of PFS in ASCT+CAR-T group. (B) Subgroup analysis of OS in ASCT+CAR-T group. In the ASCT+CAR-T group, age, disease status before treatment (SD *vs* PD), conditioning regimen before ASCT (BEAM *vs* TBC), primary or secondary CNS lymphoma, with or without systemic disease, with or without high-risk genetic abnormalities and chemosensitivity did not influence PFS and OS.


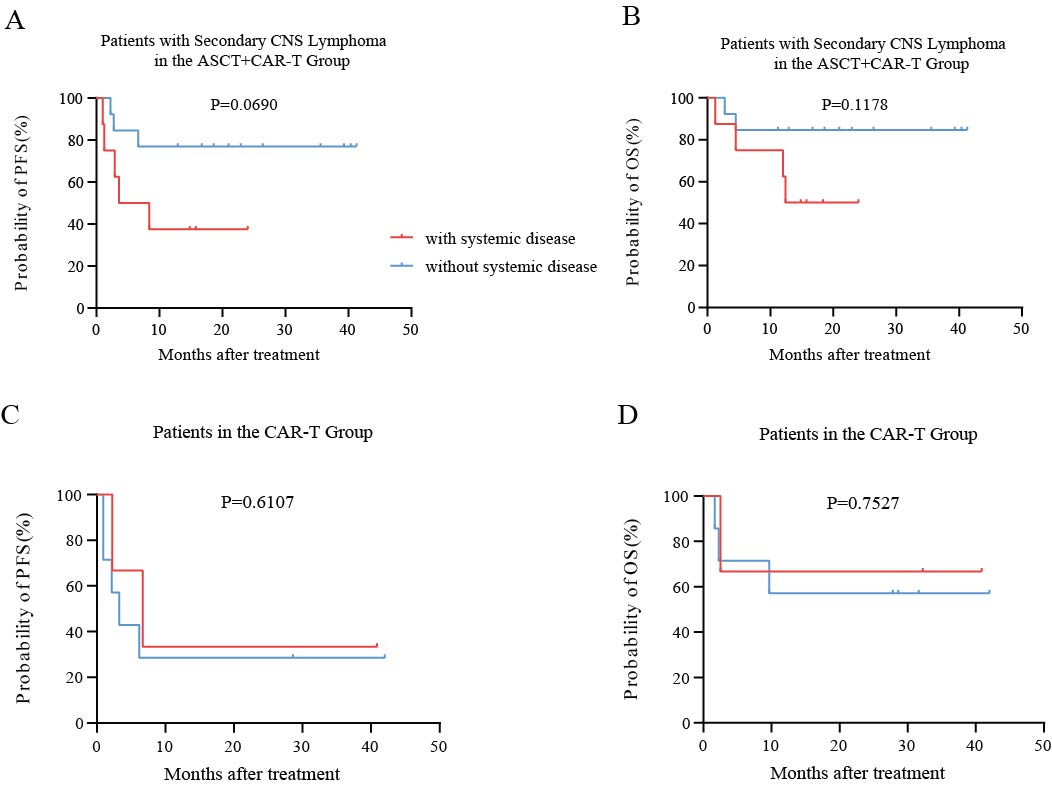


Supplementary Figure 3. Subgroup analysis of PFS or OS in patients with/without systemic disease in ASCT+CAR-T/CAR-T group. (A-B) Subgroup analysis of PFS or OS in patients with secondary CNS lymphoma with/without systemic disease in ASCT+CAR-T group. (C-D) Subgroup analysis of PFS or OS in patients with/without systemic disease in CAR-T group. The red lines represent those who with systemic disease before treatment, the blue lines represent those who without systemic disease before treatment. There was no statistically significant difference between PFS and OS of the patients with secondary CNS lymphoma with or without systemic disease in both ASCT+CAR-T group and CAR-T group.
